# Genetic and Epigenetic Aspects of Type 1 Diabetes Mellitus: Modern View on the Problem

**DOI:** 10.3390/biomedicines12020399

**Published:** 2024-02-08

**Authors:** Ildar Minniakhmetov, Bulat Yalaev, Rita Khusainova, Ekaterina Bondarenko, Galina Melnichenko, Ivan Dedov, Natalia Mokrysheva

**Affiliations:** Endocrinology Research Centre, Dmitry Ulyanov Street, 11, 117292 Moscow, Russia; khusainova.rita@endocrincentr.ru (R.K.); bondarenko.ekaterina@endocrincentr.ru (E.B.); melnichenko.galina@endocrincentr.ru (G.M.); dedov@endocrincentr.ru (I.D.); mokrisheva.natalia@endocrincentr.ru (N.M.)

**Keywords:** type 1 diabetes mellitus, *HLA*, genes, genetics, epigenetics

## Abstract

Omics technologies accumulated an enormous amount of data that advanced knowledge about the molecular pathogenesis of type 1 diabetes mellitus and identified a number of fundamental problems focused on the transition to personalized diabetology in the future. Among them, the most significant are the following: (1) clinical and genetic heterogeneity of type 1 diabetes mellitus; (2) the prognostic significance of DNA markers beyond the *HLA* genes; (3) assessment of the contribution of a large number of DNA markers to the polygenic risk of disease progress; (4) the existence of ethnic population differences in the distribution of frequencies of risk alleles and genotypes; (5) the infancy of epigenetic research into type 1 diabetes mellitus. Disclosure of these issues is one of the priorities of fundamental diabetology and practical healthcare. The purpose of this review is the systemization of the results of modern molecular genetic, transcriptomic, and epigenetic investigations of type 1 diabetes mellitus in general, as well as its individual forms. The paper summarizes data on the role of risk *HLA* haplotypes and a number of other candidate genes and loci, identified through genome-wide association studies, in the development of this disease and in alterations in T cell signaling. In addition, this review assesses the contribution of differential DNA methylation and the role of microRNAs in the formation of the molecular pathogenesis of type 1 diabetes mellitus, as well as discusses the most currently central trends in the context of early diagnosis of type 1 diabetes mellitus.

## 1. Introduction

Type 1 diabetes mellitus (T1DM) is a polygenic multifactorial disease based on immune-mediated or idiopathic destruction of pancreatic β-cells resulting in absolute insulin deficiency [1]. T1DM is the most common form of diabetes in children and adolescents worldwide [2]. In Russia, according to the federal register, as of 1 January 2023, the number of patients with diabetes mellitus registered at the dispensary was 4,962,762 people (3.31% of the population of the Russian Federation). Among them, the proportion of patients with T1DM was 5.58% (277.1 thousand), with T2DM was 92.33% (4.58 million), and with other types of diabetes was 2.08% (103 thousand) [3]. Slowly progressing T1DM in adults (late-onset autoimmune diabetes of adults (LADA)), which typically manifests itself after the age of 25 years, occurs with an incidence of from 6 to 50% in different populations. Neonatal diabetes mellitus (NDM), which manifests itself during the first 6 months of a child’s life, develops on average in 0.001% of newborns. T1DM, LADA [4], and NDM have common and specific molecular genetic markers; their identification is currently underway. The investigation of the abovementioned diabetes forms is one of the actively developing areas in fundamental diabetology [5].

In T1DM, the β-cells of the Langerhans islets in the pancreas are destroyed, which causes hyperglycemia, ketoacidosis, and a number of other concomitant disorders in the body’s homeostasis. The progress of T1DM is usually divided into three main stages. The first stage begins with the initiation of autoimmunity to β-cells and is attested by the availability of two or more islet autoantibodies with concomitant normoglycemia. The second stage is characterized by the presence of autoimmunity to β-cells and dysglycemia; this stage is presymptomatic. The third stage is the beginning of the disease itself with a complete dependence on insulin therapy [6]. Now it is recognized that the loss of β-cells may progress slowly; some people do not develop diabetes until many years after the appearance of autoantibodies. Nevertheless, in individuals of older age or in certain adults, the destruction of β-cells can begin rapidly, in contrast to a special form of slow-onset autoimmune diabetes named latent autoimmune diabetes of adults (LADA). In other words, in the common autoimmune etiology of T1DM, the rates of disease progression differ and the mechanisms underlying these differences are unknown.

T1DM has a significant hereditary risk, estimated to range from 40% to 60% based on family and twin studies; approximately 50% of this heritability is thought to be attributable to the major histocompatibility complex (HLA) region [7,8].

More than 40 years have passed since the associations of *HLA* genes with T1DM were first identified, and over this time period, researchers found that the contribution of these genes largely depends on a number of other regulatory genes and genome regions, in which the nucleotide sequence alterations play a key role in the risk of developing T1DM [5].

The National Center for Biotechnology Information (NCBI) database contains more than 20 thousand papers devoted to the investigation of genetic factors that are involved in the pathogenesis of T1DM. In addition to the genes of the major histocompatibility complex, scientific interest is focused on investigating the significance of a number of other genes such as *INS* (insulin), *PTPN22* (tyrosine phosphatase), *IFIH1* (RIG-I-like receptor), *SH2B3* (adapter protein), *CD226* (immunoglobulin superfamily protein), *TYK2* (tyrosine kinase 2), *FUT2* (galactoside-2-alpha-L-fucosyltransferase 2), *SIRPG* (signal-regulatory protein gamma), *CTLA4* (immunoglobulin superfamily protein), *CTSH* (cathepsin H), *CTLA4* (cytotoxic T-lymphocyte glycoprotein), and *UBASH3A* (ubiquitin-associated protein A containing the SH3 domain) [9]. There is evidence of augmentation in the T1DM risk through the mechanisms of genomic imprinting with the participation of the INS gene [10] and the alternative splicing of islet cell autoantigen IA-2 mRNA [11], as well as through gene–gene and gene–environment interactions stipulated by epigenetic modifications [12,13] or retrovirus-mediated changes in different cells involved in pancreatic β-cell functioning [14,15]. The most relevant areas are related to the replication of genome-wide association studies (GWAS), the analysis of rare single nucleotide polymorphic variants (SNPs) identified outside the *HLA* genes, and the investigation of epigenetic patterns in β-cells.

On the basis of the currently identified genetic predictors of T1DM, a number of mathematical models have been generated that are used as tools for predicting the risk of developing the disease. It is worth noting that all predictors included in models should have high predictive value. However, due to the lack of knowledge about the contribution of some rare genetic risk variants of *T1DM*, such as *SH2B3*, *CD226*, and *CTLA4*, prognostic models involve only the gene variants, whose changes are highly reliably associated with the mechanisms of development of islet autoimmunity and accompanied with the dysregulation of antigen-presenting cells, activation of T cell signaling, and regulation of T1 interferon levels and cytokine signaling [16]. Models are constantly improving due to the emergence of new genetic and epigenetic data.

It is obvious that the pathogenetic mechanisms of the development of T1DM have a multi-stage structure; consequently, the priority of modern medical genetics is deciphering multi-level gene interactions between the most significant elements of the disease pathogenesis. In particular, some risk genetic variants of obesity are known to be significantly associated with T1DM [5]. In addition, genes that are involved in the pathogenetic mechanisms of type 2 diabetes may play a certain role in the formation of T1DM [5]. A comprehensive analysis of DNA loci can improve both the diagnosis of T1DM [17] and the prediction of the risk of developing the disease and its complications due to the method of the polygenic risk score (PRS), which calculates an individual propensity score to a specific phenotype of a multifactorial disease based on an analysis of the contribution of a large number of loci. A separate issue in understanding the pathogenesis of T1DM is the search for the relationship between the genetic components of the disease and non-genetic factors, such as viral infections, diet, and intestinal microbiome, which potentially contribute to the T1DM etiology. In this respect, transferring research results into practice and precision medicine requires a multidisciplinary approach. Accordingly, a vast body of evidence has been obtained on hereditary factors affecting the development of T1DM, and their individual aspects will be discussed in detail subsequently.

## 2. Major Histocompatibility Complex (HLA) Locus: Population Aspects

The HLA system area is one of the most complex and polymorphic regions in the human genome and consists of more than 200 genes located within chromosome 6, p-arm at 21.3. The structure of these genes determines the individual profile and affinity of T cell receptors, which affect the functioning of the immune system. There are three classes of *HLA* genes, namely I, II, and III, differing in their functions. HLA class I molecules, in the form of transmembrane glycoproteins, are found on the surface of all nucleated cells. *HLA* genes class II are located in B lymphocytes, macrophages, dendritic cells, Langerhans islet cells, and thymic epithelial cells. The HLA class III region encodes some molecules important in inflammation including complement components C2, C4, and factor B; tumor necrosis factor (TNF)-alpha; lymphotoxin; and three heat shock proteins [18].

Genetic predisposition to the risk of developing T1DM is determined by HLA class II [19,20]. The region of HLA class II genes has a complex structure; it contains three loci, named *DR*, *DQ*, and *DP*; each of them includes a variable number of α- and β-chain genes. The *HLA-DRB* is the most polymorphic locus, which, in turn, consists of the *HLA-DRB1* gene and may also include the following genes, with dependence on the 13 gene haplotypes: *DRB3*, *HLA-DRB4*, *HLA-DRB5* and pseudogenes *HLA-DRB2*, *HLA-DRB6*, *HLA-DRB7*, *HLA-DRB8*, and *HLA-DRB9* [21].

The following three genes are of the greatest importance in clinical practice: *DRB1*, containing more than 400 allelic variants; *DQA1*, consisting of 25 allelic variants; and *DQB1*, which has 57 allelic variants. There are pronounced population differences in the frequency and spectrum of *HLA* haplotypes between world populations, as well as between European populations living in different regions of Europe. For example, in most European populations, the most common *HLA* haplotype is A*01-B*08; among the Finns, that is A*03-B*35; in populations of southeastern Europe, that is A*02-B*51 [22].

Most *HLA* risk haplotypes have been identified in cohort studies of European ancestry, and thus they may differ somewhat from those in populations of Asian and African ancestry. In particular, a comparative analysis by Harrison et al., 2020, revealed that the *DR3-DR3* variant in Indians was more significantly associated with T1DM compared to Europeans (odds ratio OR = 148.8 versus OR = 16.9 in Europeans); and, on the contrary, *DR4-DR4* was not associated with a high T1DM risk in Indians [23].

In the work by Kuraeva et al., 2017 the distribution of frequencies of *HLA* class II gene haplotypes was analyzed in Nenets and Russian populations from Moscow and the Vologda region. It is noteworthy that not a single case of T1DM has been recorded in the Nenets population over the past 50 years. Researchers found that in the Nenets population, there was a lower frequency of occurrence of two risk haplotypes, such as DRB1*17(03)-DQA1*0501-DQB1*0202 and DRB1*01-DQA1*0101-DQB1*0501, as well as higher frequency of two protective haplotypes DRB1*11-DQA1*0501-DQB1*0301 and DRB1*13-DQA1*0102-DQB1*0602/8/DRB1*13-DQA1*0103-DQB1*0602/8 compared with two groups of Russian populations; that probably contributes to low susceptibility to T1DM in Nenets [24].

Balushi et al., 2023 [25], examined the *HLA* gene profile in the population of Oman (Arabian Peninsula). The study included samples of 73 seropositive children with diagnosed diabetes (mean age 9.08 ± 3.27 years) and revealed that variants HLA B*08, B*58 class I and DQB1*02, DRB1*03, DRB1*04 class II were associated with T1DM, while B*51 and DQB1*05, DQB1*06 and DRB1*16 manifested themselves as protective alleles. The HLA-DRB1*03 and DQB1*02 alleles, as well as the heterozygous genotypes HLA-DRB1*03/*04 and DQB1*02/*03, were associated with T1DM [25].

The results of next-generation sequencing (NGS) based on DNA sampling of T1DM patients from the Laboratory of Diabetes and Inflammation (JDRF/Wellcome Trust) and control sampling from the British Cohort of 1958 Births showed that patients with the alleles DRB1*03:01 and DRB3*02:02 were at an independent risk of developing T1DM compared with carriers of the allele DRB3*01:01 [26]. Haplotypes DRB1*03:01-DRB3*02:02 have a high risk of developing T1DM (OR = 25.5, 95% CI 3.43–189.2) [26]. Another study of people of European ancestry was performed by the scientific group of Zhao, 2016. Herein, patients participated in the nationwide study “Swedish Better Diabetes Diagnosis” and were aged from 9 months to 18 years. Researchers found that among 25 alleles of the *HLA-DRB1* genes, only 4 were associated with T1DM. They were presented by DRB1*03:01:01, DRB1*04:01:01, DRB1*04:04:01 and DRB1*04:05:01. Moreover, for the *DRB4* gene, a variant DRB4*01:03:01 was associated with T1DM, while for the variant DRB4*01:01:01, no association was identified [27].

Smith et al., 2014, used NGS technology for the further sequencing of exons 2 and 3 in the *DRB1/B3/B4/B5*, *DQA1*, and *DQB1* genes and exon 2 in the *DPA1* and *DPB1* genes on the MiSeq platform. The average accuracy was 99.6%, for *DRB1* it was 97%, and for *DQB1* it was 100% [28]. It should be noted that in regard to the wide range of polymorphic variants and sequence similarities among the *HLA* genes, the use of whole genome sequencing makes accurate *HLA* mapping and typing quite challenging. However, a progressive solution to this problem was proposed by a British scientific group of Dilthey et al., 2016, in the form of the so-called population reference graph (PRG); it is made up of 46 genes and pseudogenes, mainly *HLA*, their genomic context, and described sequence variants, along with an integrated database of more than 10,000 variant alleles. This database makes it possible to bring the algorithm into effect, providing comparison reads of the *HLA* gene sequence and, as a result, infer the most likely pair of high-risk allele combinations using the likelihood principle [29].

Haris et al., 2021, examined a sampling of children and adolescents less than 18 years of age with diabetes who attended diabetes clinics or were hospitalized at Sidra Medicine, which is the only pediatric diabetes center in Qatar. They used the PRG method to identify *HLA* alleles based on exome sequencing and found that *HLA* haplotypes DQA1*03:01:01G and DQB1*03:02:01G were common in Qatari children with T1DM. These variants have been previously reported in a study of Europeans, where they were in strong disequilibrium on linkage with HLA-DRB1*4 and were significant risk factors for T1DM development [30].

Some *HLA-DQA1* variants, such as DQA1*02:01, which is protective for T1DM in European populations [5,31], are among the most common alleles in Brazilian cohorts [32]. Gomes et al., 2023, found that the risk of T1DM developing in Brazilians may be determined by risk haplotypes, which are typical to European populations; but, if the subjects were of African origin, the same risk variants had a protective effect [33].

**Figure 1:**
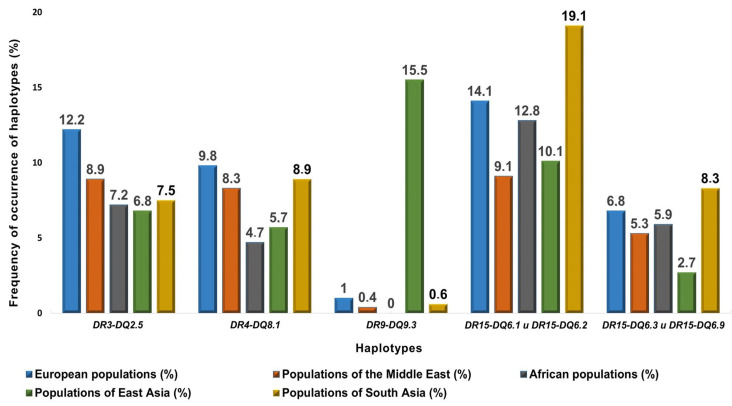
Prevalence of five known *HLA-DR-DQ* haplotype groups, involved in susceptibility/resistance to T1DM, in five different regions of the world. The drawing was created using Microsoft Excel (2016) software based on data from Redondo et al., 2022 [35].

Ethnic ancestry history affects patterns of genetic drift and selection around the world, exerting the risk of developing multifactorial diseases. Allele frequencies, including those in genomic regions, which influence the risk of T1DM, are generated by evolutionary history [34]. In particular, it is well known that genetic diversity is predominant in African populations, but due to the emphasis of modern DNA testing on *HLA* risk haplotypes specific to European populations, there is a high potential for the misidentification of T1DM risk in populations of non-European ancestry. For example, the HLA-DRB1*04:03 genotype, which is quite common among East Asians and Hispano-Americans, is protective and counteracts the high-risk alleles *HLA-DQ8* and *HLA-DQ2* attributable to Europeans. Haplotypes *HLA-DR-DQ*, which occur at low frequency in Europeans, are associated with the risk of T1DM in populations from Africa and the Middle East (Figure 1) [34].

Such differences raise the question of whether population-specific genetic associations, if they are secondary to environmental factors, may depend on the geographical location of peoples. Data indicate that the frequency of *HLA* alleles and haplotypes in European populations was generated by strong selection pressures, including the medieval bubonic plague epidemic as one of the most significant factors. In modern European populations, the HLA-DRB1*13 variant is revealed more than twice as often, while the *HLA-B* alleles encoding isoleucine at position *80 (I-80+)*, and HLA C*06:02 and *HLA-DPB1* alleles, encoding histidine at position 9, are found twice as rarely when compared to people from burials of the chronological period of the plague epidemic. Thus, significant shifts in *HLA* allele frequencies may indicate natural selection on resistance to a specific pathogen [35].

Modern research notes that over the past 50 years, the frequency distribution of HLA genotypes associated with T1DM has been significantly changed [36,37], which may indicate a shift in the processes of evolutionary selection and an increase in environmental pressure contributing to higher penetrance of the disease [38].

Thus, the investigation of genetic diversity among the different peoples of the world in terms of the prevalence of certain HLA haplotypes is of paramount importance for assessing the risk of developing T1DM.

## 3. Candidate Gene Research

The candidate gene approach allowed the identification of several genes whose changes were associated with T1DM. In particular, Bottini et al., 2004, found that the rs2476601 polymorphism in the *PTPN22* gene, encoding the lymphoid protein tyrosine phosphatase (LYP), was associated with T1DM in non-Hispanic North American Europeans; the rs2476601 polymorphism impairs the LYP–CSK complex formation, whose biological function is to inhibit T cell activation [39]. This polymorphic variant was investigated by Russian researchers as well. Ivanova et al., 2013 conducted a search on associations of the rs2476601 polymorphism with T1DM in Bashkirs, Yakuts, Buryats, Udmurts, and Russians and found that the 1858T variant of the *PTPN22* gene was associated with T1DM in the Udmurt, Russian, and Bashkir populations, while no such pattern was found in the Yakuts and Buryats [40].

Gene mapping has shown that *CTLA-4*, as well as its adjoining genes, may be involved in susceptibility to T1DM [41]. Kavvoura et al., 2005, using the MEDLINE and EMBASE databases, which contain genotyping information for 5637 people with T1DM and 6759 healthy control people, identified the A49G mutation of the *CTLA-4* gene in individuals with T1DM. Although the G allele is more common in Asian populations compared to Europeans, the risk effect associated with the presence of the G allele proved to be independent of race and ethnicity [42].

In 2007, Lowe et al., 2007, based on resequencing of the interleukin 2 alpha receptor gene *IL2RA*, identified an association between two independent groups of SNPs covering regions of 14 and 40 kilobase pairs, including intron 1 of the *IL2RA* gene and the 5′-regions of the *IL2RA* and *RBM17* genes in individuals with T1DM. Among them, rs11594656 was associated with lower circulating levels of *IL-2RA* (*p* = 6.28 × 10^−28^). The authors suggested that a genetically determined low immune response predisposes to T1DM [43].

Thus, multi-year research into candidate T1DM genes made it possible to identify several significant loci of the examined genes, which were reproduced by several researcher groups.

## 4. Multicenter and Genome-Wide Association Studies (GWAS)

Genome-wide association studies (GWAS), as well as a number of large multicenter studies, have significantly expanded knowledge about the genetic basis of T1DM; they have identified about 70 highly significant risk single nucleotide polymorphisms (SNPs) that are not localized in *HLA* genes.

Over the past 15 years, such work has been carried out by large national and international collaborations, including the Type 1 Diabetes Genetics Consortium (T1DGC) [43,44], The Environmental Determinants of Diabetes in the Young (TEDDY) [45], Diabetes Autoimmunity Study in the Young (DAISY) [46], Diabetes in the Newborn Study (BABYDIAB) [47], Wellcome Trust Case Control Consortium (WTCCC) [48], Diabetes Prevention of Type 1 (DPT-1) [5], “International TrialNet Research Network” [49,50], “Finnish Diabetic Nephropathy Study” (FinnDiane) [37], “Multinational European Project on Latent Autoimmune Diabetes in Adults” (Action LADA) [51], “Multicentre Clinical study of the Vienna Eurodiab Center” [52], etc. These studies resulted in the accumulation of an enormous amount of diverse data, much of which remain to be validated, since the ethnic component and involving different age groups might bring about rather contradictory results.

In particular, the WTCCC has identified relatively few new T1DM risk markers. Among them are the following gene variants: *ERBB3* (epidermal growth factor receptor), *SH2B3* (adapter protein), and *CLEC16A* (tyrosine phosphatase) [48]. A genome-wide association study and further meta-analysis by Barrett et al., 2009, based on a sampling of 7514 cases of T1DM and a control group of 9045 healthy individuals showed an association of more than 40 SNPs (*p* < 10^−6^) with type 1 diabetes. After excluding loci previously associated with T1DM, the other 27 loci were further examined in an independent sampling of 4267 cases of T1DM, 4463 healthy control individuals, and 2319 siblings. In GWAS replication, more than 15 loci retained a statistically significant association with T1DM (*p* < 0.01; overall *p* < 5 × 10^−8^). The most significant SNPs are localized in the interleukin genes *IL10*, *IL19*, *IL20*, and *IL27*, as well as in the transcription factor *GLIS3* gene and the cytokine *CD69* gene [53]. Mutations in the *GLIS3* gene were identified in children from three different consanguineous families with neonatal diabetes, concomitant congenital hypothyroidism, and other clinical complications [54]. The 12p13.31 region contains a number of immunoregulatory genes, including *CD69*, which is induced by T cell activation and functions in the egress of cells from the thymus; they belong to members of the family of the calcium-dependent (C-type) lectin (CLEC) domain with immune functions. The authors concluded that the relative risks for non-HLA loci were reduced in carriers of risky *HLA* haplotypes, which confirms the polygenic and genetically heterogenic structure of T1DM [55].

Despite the fact that most polymorphic variants associated with T1DM are localized in non-coding regions, it is the coding regions of DNA that are of significant interest, since they not only affect gene expression in the pancreas but also significantly change the structure of signaling proteins in immune-competent cells. Onengut-Gumuscu et al., 2015, identified coding variants associated with T1DM in seven genes including *PTPN22* (tyrosine phosphatase, previously identified in a candidate gene study), *IFIH1* (receptor of RIG-I-like receptor group), *SH2B3* (adapter protein), *CD226*, *TYK2* (Tyrosine kinase 2), *FUT2* (Galactoside-2-alpha-L-fucosyltransferase 2), and *SIRPG* (Signal regulatory protein gamma) [56].

In addition, SNPs were identified, which overlap potential enhancers next to the genes *CTLA4* (Cytotoxic T-lymphocyte glycoprotein), *CTSH* (Cathepsin H), and *UBASH3A* (Ubiquitin-associated protein A containing the SH3 domain) [56]. The authors emphasized that most markers located in enhancer sequences actively affected gene expression in thymus cells and T and B cells, as well as CD34+ stem cells. According to their preliminary inferences, enhancer–promoter interactions can now be analyzed in these cell types to determine which genes and regulatory sequences are causative, namely, determining the first links in the pathogenesis of type 1 diabetes.

It is worth noting that the rs2476601 loci of the *PTPN22* gene and rs11203203 of the *UBASH3A* gene are associated with the emergence of autoimmunity to pancreatic β-cells, while polymorphic variants of the *INS*, *UBASH3A*, and *IFIH1* genes are associated with the transition from an autoimmune reaction against pancreatic β-cells to the development of clinical diabetes. Similar results were obtained in participants in the TEDDY study, where carriers of high-risk *HLA* haplotypes and four risk polymorphic variants including rs2476601 in *PTPN22*, rs2292239 in *ERBB3*, rs3184504 in *SH2B3*, and rs1004446 in *INS* exhibited a significant association with the development of an autoimmune reaction to β-cells of pancreatic gland islets [57]. The Finnish Childhood Diabetes Registry revealed that the *DR3-DQ2/DR4/DQ8* genotype affected the production of islet β-cell autoantibodies but not the subsequent development of T1DM [58].

In the BABYDIAB study, children of parents with T1DM are under control since their birth. In this study, a risk scoring model, developed on the basis of eight loci including *IFIH1*, *CTLA4*, *PTPN22*, *IL18RAP*, *SH2B3*, *KIAA0350*, *COBL* and *ERBB3* could predict T1DM in children with high-risk *HLA* genotypes [59]. The BABYDIAB researchers also developed a criterion that used odds ratios for polymorphic variants on weights (effects) and included HLA haplotypes as an addition to nine variants in the *PTPN22*, *INS*, *IL2RA*, *ERBB3*, *ORMDL3*, *BACH2*, *IL27*, *GLIS3*, and *RNLS* genes [60]. This 10-factor score was tested to predict T1DM in two DAISY cohorts, namely among first-degree relatives, where it was more predictive than a combination with fewer SNPs, and in the general population, where it did not have any advantages over the three-factor model [61].

In 2021, Kim et al., 2021, published the results of a comprehensive analysis of T1DM based on a GWAS that identified pathogenic enhancer sequence changes in target genes. The most significant were rs886424, rs3129716, rs1264361, and rs9268606 in the region of the *HLA* gene and rs4788084, rs62031562, rs743590, and rs762633 in the 16p11.2 locus. Furthermore, 114 SNPs with high-risk T1DM, including 24 genes, specifically 6 *HLA* genes and 18 non-*HLA* genes, overlap with 159 genes detected by the eQTL (quantitative trait loci) analysis. Fifty-four percent of them, namely 13 of 24 genes, identified by the Contextualization of SNPs in the Three Dimensions (CoDeS3D) system, were involved in the immune response. In addition, 78 loci were found in 42 genes of long non-coding RNA; 13 of these 78 loci were located in enhancer regions, and among them, rs3129716 and rs886424 were highly likely to be the most significant enhancer SNPs. The authors inferred that most of the identified target genes played an important role in the immune response and regulation of activity of gene expression including *HLA* genes (6p21) [62].

Genome-wide association studies and their meta-analysis, as well as their replication and bioinformatics processing, have highlighted an enormous number of previously unknown DNA markers, which confirmed the complex heterogeneous and polygenic structure of T1DM. Currently, it is a relevant issue to assess the contribution of the enormous number of variants with a small risk effect (odds ratio (OR)), located throughout the genome, in the heterogeneity of the disease and clinical outcomes of T1DM.

## 5. The Polygenic Risk Score in Individuals with Type 1 Diabetes Mellitus

One of the promising methods of bioinformatics analysis, which helps to assess the hereditarily determined risk of multifactorial diseases, is the polygenic risk score (PRS), which implies calculating an individual susceptibility coefficient to a specific phenotype of disease on the basis of the analysis of a large number of polymorphic variants. An obvious application of PRS in the diagnosis of T1DM is assessing the risk contribution of the complex combined effects of *HLA* risk haplotypes jointly with other DNA markers. The assessment is performed on the basis of a polygenic score, which is calculated using a weighted sum of individual risk alleles significantly associated with the trait [63].

Winkler et al., 2014, using multivariate logistic regression of data (sampling N = 5781) from the Type 1 Diabetes Genetics Consortium (T1DGC), found that the additional inclusion in the model of 40 SNPs of genes, which are not located in the *HLA* locus, significantly improved the prediction of T1DM, compared with models that include only *HLA* alleles and haplotypes. On the basis of the method of including and excluding genetic predictors, the authors selected a model with optimal predictive value which involved the following genes: *HLA*, *PTPN22*, *INS*, *IL2RA*, *ERBB3*, *ORMDL3*, *BACH2*, *IL27*, *GLIS3,* and *RNLS* (AUC = 0.86, 95% CI 0.84–0.88) [64].

Oram et al. developed a polygenic risk model for T1DM in young individuals (20 to 40 years), which included 30 SNPs using the “weights” (OR for each SNP) of the *HLA DR3* and *DR4-DQ8* genes from the Winkler study [64], but they used a special “tag” of SNPs for key alleles of HLA genes and assessed to what extent the PRS model distinguished groups of patients with type 1 and type 2 diabetes. The results showed the high sensitivity and specificity of the model for predicting T1DM (AUC = 0.87, 95% CI: 0.82–0.92, *p* < 0.0001), significantly differentiating this sample from young people with type 2 diabetes. Furthermore, the model’s ability to distinguish these two groups was based on the variants *DR3/DR4-DQ8*, *DR3/DR3*, *DR4-DQ8/DR4-DQ8*, *DR4-DQ8/X DR3/X*, *DRB1_15*, *PTPN22* (rs2476601), *INS* (rs689), *IL2RA* (rs12722495), and *ERBB3* (rs2292239). The authors inferred that the combined PRS model provided the best recognition of the progression of severe insulin deficiency compared with conventional clinical predictors and biomarkers [65].

In a study by Bonifacio et al., 2018, genetic risk scores for T1DM were calculated for more than 3000 children with no family history of type 1 diabetes but having one of the two highest-risk *HLA* genotypes, namely heterozygous *DR3* and *DR4-DQ8* or homozygous *DR4*-*DQ8*, with the involvement of other DNA loci. The model has been proven to identify up to 25% of future childhood cases of T1DM. Thus, genetic models derived from the score of multiple risk loci may improve risk stratification of presymptomatic T1DM [66].

In light of the results described above, DNA screening for identifying groups with a high risk of T1DM has become important. Screening for T1DM using a variety of diagnostic tests at multiple time points during the first years of a person’s life is feasible but quite expensive. This problem could be solved with DNA analysis, which does not depend on age and environmental factors and does not change over time, so the introduction of PRS disease prediction models in newborn screening could identify individuals at high risk for broader monitoring and prevention of severe consequences of T1DM.

## 6. Functional Role of DNA Risk Loci Related to Developing T1DM in Signaling Pathway Changes

**Figure 2:**
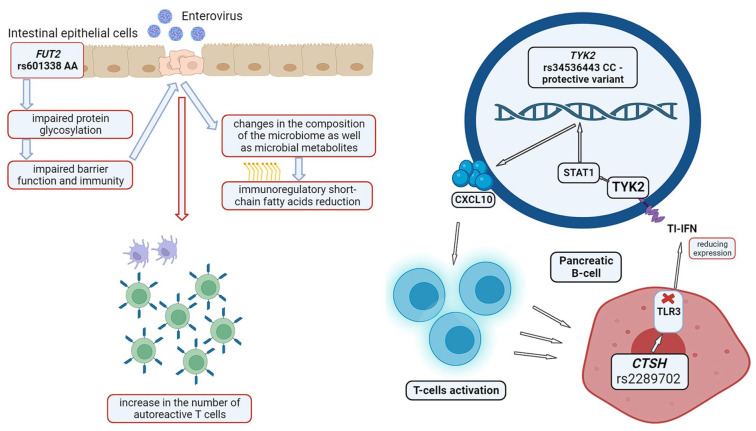
Illustration of the impact of polymorphic loci of the *FUT2*, *TYK2*, and *CTHSH* genes on autoimmunity signaling in type 1 diabetes mellitus. The drawing was created using BIORENDER web-tool based on data from Shapiro et al., 2021 [67].

**Figure 3:**
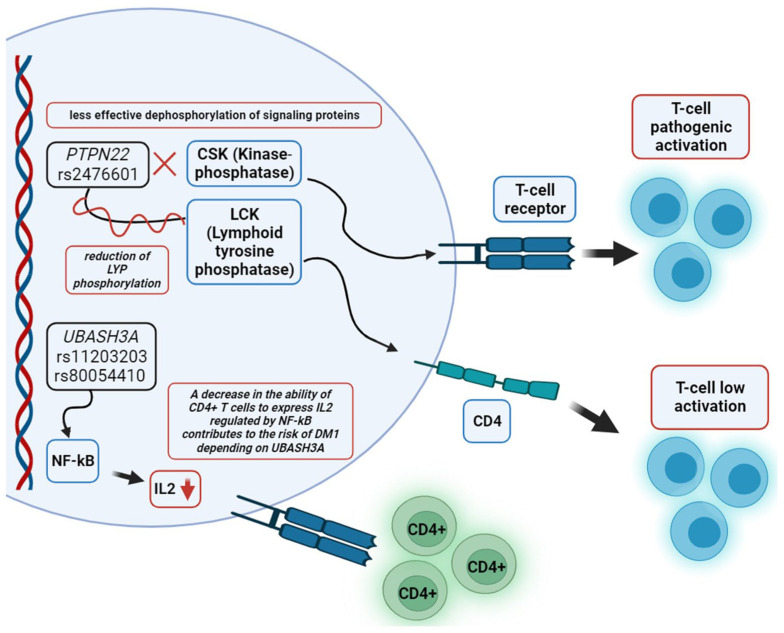
Effect of polymorphic loci of the *PTPN22* and *UBASH3A* genes on autoimmunity signaling in T1DM. The red arrow indicates a decrease in the level of expression. The drawing was created using BIORENDER web-tool based on data from Suomi et al., 2021 [81].

Once DNA loci have been identified, the next step should be directed to the investigation of their role in the functional and molecular alterations in cell signaling pathways that bring about T1DM development (Figure 2 and Figure 3). In the study by Shapiro et al., 2021, the researchers suggested that the dysfunction of FUT2 (galactoside-2-alpha-L-fucosyltransferase 2), due to gene mutation, leads to a lack of secretion of the ABO blood group antigen through the intestinal mucosa. That, in turn, may cause an impairment in the immune barrier of intestinal epithelial cells, which results in increased susceptibility to certain viral infections, as well as in changes in the composition of the microbiome and microbial metabolites, especially short-chain fatty acids. The expression of the ABO antigen in the intestinal mucosa affects the binding of exogenous pathogens and commensal microbiota. The fecal microbiota of individuals with the rs601338*A/A variant was found to contain, on average, fewer probiotic bifidobacteria, which are capable of producing immunoregulatory short-chain fatty acids and promoting intestinal barrier integrity, which is critical for preventing commensal-induced autoimmunity [67]. The rs601338 risk allele is associated with a sharp deterioration in the first phase of insulin response in children with multiple autoantibodies at T1DM [67]. This fact could explain the relationship between patient age and secretory status at the time of diagnosis. Examinations indicated that therapy, positively affecting FUT2, was likely to be required at an early age for patients with a specific genotype [68,69].

In the context of the pathogenesis of T1DM, TYK2 (a member of the JAK family) enhances antigen presentation by stimulating the expression of *HLA* gene class I and promotes the expression of the chemokine CXCL10, which causes the activation of T cells and their recruitment toward the pancreatic islets, thereby increasing the risk of developing the autoimmune process [70] (Figure 2). Moreover, this effect may be significantly complicated by the biological activity of Cathepsin H (CTSH). Shapiro et al., 2021, made a number of assumptions regarding the importance of this molecule. The fact is that CTSH is a lysosomal proteinase, which plays a role in protein recycling, prohormone processing, and HLA II antigen presentation, as well as it may antagonize CXCL10.

Even as CTSH is expressed ubiquitously, its representation is most pronounced in type II lung alveolar cells during the maturation of surfactant protein [71]. Allele C of the rs2289702 locus in exon 1 of the *CTSH* gene turned out to be protective at T1DM; it can affect the cleavage of cathepsin to its active form and its delivery to lysosomes [72]. The T allele of this locus is associated with the prevention of an early onset of T1DM, especially in patients younger than seven years [73]. One possible explanation for this finding is that decreased *CTSH* gene expression may reduce the N-terminal cleavage of Toll-like receptor 3 (TLR3), impairing TLR3 functionality and dropping TI-IFN expression in response to viral infections in early childhood [73]. However, insight into the mechanism of the relationship between *CTSH* expression and the risk of developing T1DM gets further complicated by the report of *CTSH* overexpression, which induces intrinsic β-cell protection from cytokine-mediated damage and the stimulation of insulin production [74] (Figure 2). Functional examinations support the relevancy of continuing investigations of CTSH modulation as a potential means of preventing T1DM with specific attention to the off-target effects of targeted therapy toward this protein expression in the treatment of T1DM.

The polymorphic variant rs2476601 in exon 14 of the *PTPN22* gene leads to the replacement of arginine with tryptophan at position 620 and is one of the loci most significantly associated with T1DM, being second in importance only to the *HLA* and *INS* variants. The gene encodes non-receptor lymphoid tyrosine phosphatase type 22 (LYP), which is responsible for dephosphorylation of signaling proteins. LYP is one of the most powerful inhibitors of T cell activation. The substitution affects the interaction between the LYP proline-rich motif and CSK tyrosine kinase, causing the impairment of signal transduction modulation. The study indicates that the mutation is associated with the synthesis of autoantibodies to insulin, which manifests itself more rapidly in children carrying high-risk *HLA* haplotypes or in first-degree relatives with type 1 diabetes [75]. Nevertheless, the role of rs2476601 in enhancing T cell activity remains controversial, since there is uncertainty in understanding how this variant affects the functional activity of tyrosine phosphatase [76]. However, it is reliably known that the rs2476601 polymorphism impairs the interaction between LCK and LYP, which is accompanied by a decrease in LYP phosphorylation and, ultimately, contributes to the inhibition of gain in the function of T cell activation [77] (Figure 3). It is believed that a gain in LYP activity may be a predisposition to autoimmunity through the decreased activation of regulatory T cells, which are required to suppress autoreactivity [78]. When regarding a potential complete loss of tyrosine phosphatase activity, it implies impairment of the signaling apparatus of T cell receptors, which, in turn, leads to the less effective dephosphorylation of signaling proteins and increased activation of effector T cells [79].

T cell ubiquitin-1 ligand UBASH3A reduces T cell receptor signaling. Currently, most T1DM-associated variants of the *UBASH3A* gene are intronic. Meanwhile, it is known that UBASH3A regulates the NF-κB signaling pathway through a ubiquitin-dependent mechanism and that the risk alleles rs11203203 and rs80054410, associated with T1DM, increase the expression of the *UBASH3A* gene in primary human CD4+ T cells upon the stimulation of T cell receptors, which results in reducing NF-κB signaling through the IκB kinase complex and diminishing *IL2* gene expression [80] (Figure 3). Suomi et al., 2023, published a summary of the results of transcriptomic profiling of whole blood samples from patients with T1DM as part of the INNODIA study. They found that the expression of some genes and the activity levels of signaling pathways involved in innate immunity were reduced during the first year after diagnosis. A significant change in gene expression was associated with positive ZnT8A autoantibody status [81].

On the other hand, *SIRPG*, *STXBP1*, and *UBASH3A* genes had an inverse correlation with positive ZnT8A autoantibody status. SNPs, associated with T1DM, near the *SIRPG* gene have been shown to modulate disease risk by controlling the alternative splicing of the gene. It encodes syntaxin binding protein 1, which regulates the docking and fusion of vesicles with the plasma membrane during exocytosis. STXBP1 is important for the cytotoxic activity of CD8+ T cells and NK cells. The *UBASH3A* genetic variant is associated with the development of T1DM in children from the DAISY and BABYDIAB cohorts. Type 1 diabetes-associated variants of the human *UBASH3A* gene caused higher levels of gene expression and decreased NF-κB signaling and *IL2* expression in CD4+ T cells [81].

## 7. Molecular Pathogenesis of Autoimmune Diabetes in Adults

Latent autoimmune diabetes in adults (LADA) is based on pathogenetic processes attributed to both T1DM and T2DM. It includes an autoimmune reaction with the simultaneous involvement of adaptive and innate immunity, as well as, to a lesser extent, the development of insulin resistance and metabolic syndrome [82]. LADA is also characterized by an older age of manifestation, from 25–35 years, as well as a slower rate of β-cell destruction when compared to T1DM. Despite the detection of autoantibodies to β-cells, there are certain differences between T1DM and LADA. In T1DM, as a rule, all variants of autoantibodies were detected (ICA, GAD, IA-2, IAA), while in LADA, only one or two variants, mainly GAD and ICA, were identified; IA-2 and IAA were found extremely rarely [83]. Genetic studies have revealed that the hereditary component of LADA contains the same DNA markers as at T1DM. They include risk haplotypes of the *HLA* genes, in particular *HLA-DRB1*, *HLA-DQA1*, and *HLA-DQB1* genes, as well as variants of the *PTPN22*, *INS*, and *CTLA-4* genes [82].

The haplotypes DRB1*0405-DQA1*03-DQB1*0401 and DRB1*0901-DQA1*03-DQB1*0303 have the strongest associations between the *HLA* complex and LADA. They are also associated with the risk of T1DM (OR = 2.02 (LADA), OR = 2.20 (T1DM) and 1.61 from 2.30, respectively). Moreover, the haplotype DRB1*0301-DQA1*05-DQB1*0201 is also associated with LADA; however, the OR value is half that of T1DM (2.65 versus 4.84) [82]. Currently, common risk loci for LADA and T1DM are known; they are localized in the *CTLA-4* gene and include rs2033171, rs3087243, rs231806, rs231775, rs5742909, and rs16840252. Examining the role of this gene in autoimmune processes at LADA is of scientific interest, given the high variability in the *CTLA-4* gene and the diversity of isoforms of its protein product [82].

In 2018, the first results of a genome-wide association study of LADA were published; they detected the risk polymorphic variants inherent to T1DM (*HLA*, *PTPN22*, *INS*, etc.) and T2DM (THADA) and coupled with the disease. In addition, GWAS revealed a new independent association in the known rs1983890 locus of the *PFKFB3* gene, encoding a protein involved in the regulation of glycolysis and insulin signaling in T2DM [83]. The authors inferred that LADA has a genetic component, which enhances autoimmune processes, and a component associated with metabolic syndrome at T2DM. The findings also support the hypothesis that the LADA polygenic structure, related to T2DM susceptibility, may act as a risk modifier of T1DM in patients with moderate autoimmune susceptibility. However, it is insufficient to cause T1DM in children but is sufficient to cause predisposition to LADA. Thus, based on the current knowledge, LADA can be described as a hybrid form of T1DM and T2DM, manifesting phenotypic and genotypic similarities with both forms [83,84]. Table 1 presents the DNA loci most significantly associated with LADA, which were reproduced in different studies [83].

## 8. Epigenetic Factors in Type 1 Diabetes Mellitus

Epigenetic factors are involved in the fulfillment of genetic information encoded in DNA sequences by mechanisms that do not affect the nucleotide sequence. Epigenetic changes are preserved in a number of mitotic divisions of somatic cells and can also be transmitted to subsequent generations. They are dynamic and reversible and can change under the effect of environmental factors. The following types of epigenetic mechanisms are known: chromatin remodeling, RNA interference, and DNA and RNA methylation [85].

Currently, specialists know much about the genetic causes of T1DM, while the epigenetic mechanisms and their aberrations underlying the pathogenesis of the disease have been little investigated. However, certain progress has been made in examining the DNA methylation profile in patients with T1DM. DNA methylation is one of the key mechanisms for regulating gene transcription activity, embryonic development, modification of chromatin structure, X-chromosome inactivation, as well as chromosomal stability and genomic imprinting. This process is a form of covalent modification of DNA without changing its sequence, which provides the transfer of a methyl group (CH3) from S-adenosylmethionine to position 5 of the pyrimidine ring of cytosine. Typically, gene methylation causes a decrease in gene expression; however, the effect may vary depending on the methylation of individual CpG sites within or outside the regulatory regions of the gene [86].

In 2011, Rakyan and colleagues published the data of an epigenome-wide association study (EWAS) on the DNA methylation profile of purified CD14+ monocytes in 15 pairs of twins discordant on T1DM by sampling from the British Diabetes Twin Study. Analysis of the results made it possible to identify 132 differentially methylated CpG sites, which correlated with T1DM and included 58 hypermethylated and 74 hypomethylated ones. This group involved the following genes: *HLA-DQB1* with a high level of significance; GAD65 relevant to the main autoantigen in T1DM; *TNF*, encoding a key pro-inflammatory cytokine; and *TRAF6*, encoding one of the proteins from the Toll-like receptor signaling pathway. The authors emphasized that differential methylation levels in twins indicated epigenetic changes that were associated with an early stage of the etiological process of T1DM [87]. Further, their findings were partly confirmed in the study conducted by Starskaia and colleagues. In 2022, they assessed early changes in DNA methylation, associated with T1DM, before the diagnosis or before the emergence of autoantibodies in children who later developed islet autoimmunity at a young age. For this purpose, they separated CD4+, CD8+ and CD4-CD8 T cell subpopulations and determined differences in DNA methylation levels among specific cell types. In addition, to identify DNA methylation changes associated with T1DM, they examined correlations between DNA methylation and gene expression. Eventually, the analysis of fractionated samples allowed them to detect DNA methylation changes specific to immune cell subpopulations. Changes were revealed in genes associated with T1DM, including *TRAF3* (TNF receptor-associated factor), *DGKQ* (Diacylglycerol kinase), and *IL32*, as well as novel genes such as *ARRDC2* (arrestin domain containing 2) and *PCBP3* (Polybinding protein). In addition, they confirmed the results using pyrosequencing at the following CpG sites: chr19:18118304 in the *ARRDC2* promoter; chr21:47307815 in the *PCBP3* intron; and chr14:81128398 in the intergenic region near *TRAF3* in CD4+ T cells [88].

In 2014, Olson et al. reported data on genome-wide DNA methylation in human pancreatic islet cells that identified 383 CpG sites with high levels of association after adjusting for multiple testing. These sites were located in the following genes: *ADCY5* (Adenylate cyclase type 5), *KCNJ11* (potassium inward rectifying channel subfamily J member 1), *HLA-DQA1*, *INS*, *PDX1* (insulin promoter factor 1), and GRB10 (Growth factor receptor-bound protein 10) [89].

In 2016, Paul et al., 2016, studied the methylation profile of DNA in immune cells from 52 T1DM discordant twin pairs on the Illumina HumanMethylation450 BeadChips platform and identified a significant number of differentially methylated CpG sites in T1DM twins compared to healthy identical twins and a healthy control group. Further analysis revealed that associated CpG sites were located on regulatory elements of genes involved in immune cell metabolism and the cell cycle, such as mTOR signaling [90]. The most significant association was shown by the cg01674036 locus, detected in the promoter region of the *DDIT4* gene, encoding the protein inhibitor of rapamycin complex 1 (mTOR), which is involved in the regulation of T cell activity [90].

In a study by Johnson et al., changes in DNA methylation profiles were assessed in blood samples collected before T1DM diagnosis in children enrolled in the prospective DAISY cohort using the Illumina 450 K and EPIC platforms [91]. They revealed 28 differentially methylated CpG sites associated with the later development of T1DM. Hypermethylation averaged 4.1% compared to controls and was most significant at site cg24891731 (*p* = 1.49 × 10^−7^). It was detected next to the processed transcript *CTD-2281E23.2* within chromosome 8, next to the *ERICH1-AS1* gene. The cg11405300 locus with a significance level of 7.16 × 10^−7^ was characterized by hypermethylation of 3.1% compared to the control and was detected next to AP000911.1, a new microRNA within chromosome 11 [91].

Data indicate that the risk of T1DM may be mediated by microRNAs, which are combined into a group of small non-coding RNAs up to 20 nucleotides in length and involved in the regulation of gene expression at the transcriptional or post-transcriptional levels. Most studies measuring miRNA levels in T1DM have been conducted in vitro and in vivo in mouse strains, as well as in human plasma. One such example is the overexpression of microRNA-21 in β-cells of the NOD mouse strain with T1DM and in the blood serum of 19 children at the time of T1DM diagnosis; the mechanism was based on increased β-cell apoptosis due to a specific effect on the translation of the *BCL-2* gene [92]. The study, conducted in children from a Danish cohort, revealed that the expression of microRNA-181 was increased in T1DM patients when compared with healthy controls. The correlation of the level of this microRNA with the levels of C-peptide and SMAD7 identified it as one of the pathogenetic factors of β-cell dysfunction [93]. Interleukin IL-1b and TNF-a increased the expression of microRNA-21, microRNA-34, and microRNA-146, while the levels of microRNA-23 and microRNA-149 decreased with the participation of IL-1b and interferon followed by the activation of β-cell apoptosis [94].

Some microRNAs impair the pathways of biosynthesis and glucose-stimulated insulin secretion. One of them is microRNA-375, which is found in high concentrations in cells of pancreatic islets and reduces glucose-induced insulin secretion by affecting PDK1 kinase, as well as genes related to insulin exocytosis, such as AIFM1, MTPU, GPHN, and YWHAZ. Furthermore, microRNA-9 and microRNA-30 similarly affect the expression of the INS gene, by reducing the expression of the MAP4K4 gene, or by decreasing the expression of the transcription factor genes: MAFA and ONECUT2. MicroRNA-25 is associated with the risk of T1DM, since it interacts with the insulin gene [95]. MicroRNA-126 has been found to correlate with retinopathy in T1DM. Expression levels of microRNA-144 are associated with diabetic heart disease, the production of reactive oxygen species, and subsequent cell apoptosis; this microRNA is significantly associated with the production of the inner mitochondria membrane phosphate transporter in cells of diabetic heart, directly affecting the production of mitochondrial ATP [96].

It has been suggested that the risk of developing T1DM may be closely associated with changes in histone deacetylase (HDAC) activity, as HDACs have been found to affect innate and adaptive immunity. However, the mechanisms of this process are poorly understood. Histone H3 was found to be hyperacetylated in the promoter regions of the TNFα and COX-2 genes in monocytes isolated from patients with T1DM. Miao et al., 2012, published the results of profiling post-translational modifications of histone regulatory genes associated with DM1 and found significant changes in H3K9Ac levels in the upstream regions of *HLA-DRB1* and *HLA-DQB1*. Additional analyses performed with mononuclear cells showed increased expression of *HLA-DRB1* and *HLA-DQB1* genes and changes in H3K9Ac levels on the background of interferon-γ and TNF-α treatment [97]. Increased acetylation levels affecting the function of these genes are associated with increased transcriptional activity in the monocyte cell line. However, it is still too early to conclude whether these discrepancies are the causal factor of the disease or the result of hyperglycemia [98].

The apoptosis of β-cells underlies the progression of T1DM, and the anti-apoptotic protein BCL-XL plays one of the key roles in this process [99]. A study by Hu et al. showed that HDAC3-mediated increase in *BCL-XL* expression through the negative regulation of the miR-296-5p gene promoter inhibits lymphocyte apoptosis, increasing the risk of T1DM development [100]. The examination of proinflammatory mediators such as COX-2 in monocytes showed an increase in the level of acetylated histone H4 in patients with T1DM [101].

The disruption of insulin signaling, leading to insulin resistance, can be put into action at several levels of regulation involving histone deacetylases. HDAC1 and HDAC4 are known to be negative regulators of expression of the *GLUT4* gene, which encodes glucose transporter type 4, an insulin-dependent glucose transporter protein. In addition, HDAC2 and HDAC5 may also be potential regulators of GLUT4. Consequently, HDACs may act as a target for the treatment of insulin resistance in muscle tissue, as compensatory transcription of GLUT4 may reverse the state of insulin resistance. Thus, our results infer that histone deacetylases play a significant role in the pathogenesis of T1DM and may also be promising targets for therapy of the disease [102].

The potential for using microRNAs in targeted therapy for T1DM has certain prospects, which is evidenced by a number of studies indicating their important role in the pathogenesis of the disease. Although the results are encouraging, much more research and clinical trials are required to promote the most effective ways to develop therapeutic agents based on the epigenetic regulation of the pancreatic β-cell function. The most significant differentially methylated CpG sites, which are associated with T1DM, differ within individual EWAS findings. Epigenetic patterns of DNA methylation are reversible and dynamic and can be affected by a significant number of factors, including environmental factors, stages of cellular development, grouping in different CD+ cell subpopulations, age, gender, and many other factors. Although markers of differential methylation status have not yet been replicated, certain progress was made in the search for potential epigenetic markers of T1DM. Based on the available data, the most likely candidates may be differentially methylated DNA regions localized within the *HLA-DQB1* and *DQA1* genes, as well as the *TRAF6* and *TRAF3* genes, which play one of key roles in autoimmune and inflammatory processes; the role of other genes is less obvious. Currently, epigenomic investigations on T1DM are in their early stages, but the findings are promising and expand fundamental knowledge about the molecular pathogenesis of the disease.

## 9. Prospects for Investigation of the Genetics of Type 1 Diabetes Mellitus

**Figure 4:**
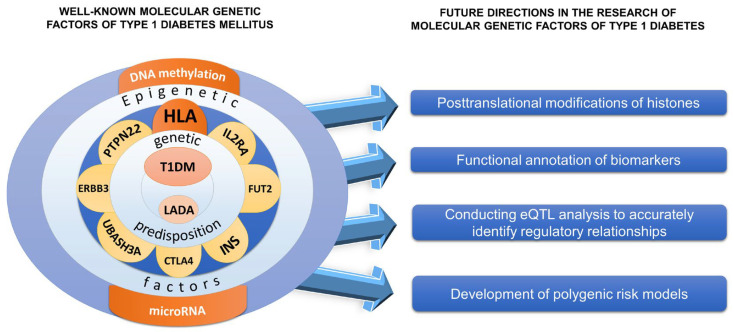
Prospects for investigation of the genetics of type 1 diabetes mellitus. The drawing was created using Microsoft Power Point software (2016).

Some of the most important areas of modern diabetology are the investigation of the molecular profile of patients with T1DM based on genetic, epigenetic, and bioinformatic analysis; the correlation of the data obtained with gene expression, microRNA, DNA, and RNA methylation (QTL); and the detection of new diabetes markers for the most socially significant complications, such as diabetic ketoacidosis, cardiovascular alterations, nephropathy, retinopathy, cognitive impairment, and diabetic limb amputations, in order to identify the molecular genetic basis of the heterogeneity of the disease, as well as to develop effective methods for the prevention and treatment of this disease, considering the population characteristics of patients around the world (Figure 4).

The main directions for the further investigation of the molecular genetic basis of T1DM:

- Wide use of genome-wide association study analysis, genome-wide differential methylation analysis based on high-throughput parallel DNA sequencing to examine the disease in different populations and regions of the world;
- Development of new and selection of modern bioinformatics tools to search for biomarkers and perform geno-phenotypic correlations of the qualitative and quantitative features of T1DM;
- Replication of the most significant results that determine an increased susceptibility to the development of T1DM in independent samples, considering their ethnic origin;
- Functional annotation of the identified biomarkers of T1DM;
- Performing eQTL (expression quantitative trait loci) analysis for accurate detection of regulatory intercommunications and determining the significance of loci in the regulation of driver gene activity and gene expression.

The use of genetic, epigenetic, and metabolomic data from thousands of patients in conjunction with information about environmental factors and the lifestyle of patients will create the basis for a unique and transcendent method for predicting the risk of developing T1DM, its personalized prevention, as well as for the targeted therapy of the disease and its complications.

## 10. Conclusions

Recent investigations have shown that genetic mechanisms play a key role in the etiology and pathogenesis of T1DM. An enormous amount of scientific data have been accumulated on the genetic factors affecting the development of the disease. However, the genetics of T1DM are complex and polygenic. Owing to a comprehensive analysis of the genetic factors of T1DM, investigation on the expression of loci of quantitative traits has been carried out; in particular, the role of genetic loci that determine differences in mRNA expression levels was described. In addition, the analysis of SNP haplotypes allowed expanding knowledge on the etiology and pathogenesis of T1DM at the level of not only structural impairments of key proteins but also at the level of their quantitative changes. To date, the relationship of risk DNA markers with the regulation of the activity of driver genes and the expression of regulatory genes that play a key role in the pathogenesis of T1DM have been poorly examined. In addition to genetic factors directly involved in the control of the immune response and β-cell functioning, other pathogenic mechanisms may be involved, as evidenced by a large body of evidence. The majority of GWAS risk variants are located in noncoding regions of the genome, suggesting that changes in gene regulatory regions significantly contribute to the development of T1DM. However, identifying causal regulatory variants associated with T1DM risk and their target genes is challenging due to the lack of knowledge about non-coding regulatory elements as well as the cellular processes in which they are involved. An additional complication lies in racial/ethnic differences, since most current knowledge comes from investigations in European populations, despite the fact that T1DM occurs in virtually all racial/ethnic groups. The most important practical application of the results of genetic research is the improvement of the prediction of diabetes in order to develop and implement strategies for preventing the disease in individuals at a high risk of T1DM. Furthermore, genetic testing may play a unique role as an independent tool for diagnosing different types of diabetes and autoimmune diseases, as well as for determining clinical outcomes in individuals with type 1 diabetes. It is hoped that advances in the investigation of T1DM genetics will make it possible to identify DNA markers with high prognostic significance that will allow them to be used in clinical practice for personalized early diagnosis and treatment of the disease, based on the principles of precision medicine.

## Figures and Tables

**Table 1:** Characteristics of polymorphic loci associated with LADA.

Loci	Gene	Odds Ratio and Condifedence Interval	*p*
rs9273368	*HLA-DQB1*	3.12 (2.86–3.40)	7.9 × 10^−143^
rs2476601	*PTPN22*	1.62 (1.48–1.78)	<0.0001
rs689	*INS*	1.39 (1.29–1.48)	<0.0001
rs7310615	*SH2B3*	1.28 (1.19–1.38)	4.9 × 10^−11^
rs7903146	*TCF7L2*	1.19 (1.00–1.40)	0.04
rs1983890	*PFKFB3*	1.16 (1.14–1.32)	3.0 × 10^−8^
